# Comparison of intracardiac vs. transesophageal echocardiography for “one-stop” procedures of combined radiofrequency catheter ablation and left atrial appendage closure with the Watchman device in the treatment of atrial fibrillation

**DOI:** 10.3389/fcvm.2023.1265550

**Published:** 2023-11-01

**Authors:** Xining Shang, Mingyu Sun, Zulu Wang, Zhiqing Jin, Ming Liang

**Affiliations:** ^1^ National Key Laboratory of Frigid Zone Cardiovascular Diseases (NKLFZCD), General Hospital of Northern Theater Command, Shenyang, China; ^2^ Department of Cardiology, General Hospital of Northern Theater Command, Shenyang, China; ^3^ Department of Cardiology, General Hospital of Northern Theater Command of China Medical University, Shenyang, China

**Keywords:** atrial fibrillation, intracardiac echocardiography, transesophageal echocardiography, catheter ablation, left atrial appendage closure

## Abstract

**Background and objective:**

This study aimed to assess the efficacy and safety of “one-stop” procedures that combined radiofrequency catheter ablation and left atrial appendage closure (LAAC) with the Watchman device under the guidance of intracardiac echocardiography (ICE) vs. transesophageal echocardiography (TEE) in patients with atrial fibrillation.

**Methods and results:**

In this study, we prospectively enrolled patients who underwent “one-stop” procedures under the guidance of ICE (*n* = 193, 109 men, 65.02 ± 8.47 years) or TEE (*n* = 109, 69 men, 64.23 ± 7.75 years) between January 2021 and October 2022. Intraprocedural thrombus formation in the left atrial appendage (LAA) was observed in 3 (1.46%) patients in the ICE group and 15 (11.63%) patients in the TEE group (*P* < 0.05) before LAAC. Total fluoroscopy time and dose in the ICE group were less than those in the TEE group. The total “one-stop” turnaround time and LAAC procedure time in the ICE group were significantly shorter than those in the TEE group (*P *< 0.05). Postoperative esophagus discomfort, nausea and vomiting, and hypotension were more often seen in the TEE group (*P* < 0.001). There was no significant difference in matched cases between ICE and fluoroscopy measurements (*P* = 0.082). The TEE results related to LAAC and clinical events were similar between the two groups during the follow-up (*P* > 0.05).

**Conclusion:**

The ICE-guided “one-stop” procedure was safe and feasible with less radiation exposure, shorter turnaround time, and fewer complications and intraoperative thrombus formations than the TEE group. ICE offered accurate measurements in the LAA dimension during LAAC. Echocardiography during the “one-stop” procedures was necessary to rule out the intraoperative thrombus.

## Introduction

Left atrial appendage closure (LAAC) has been an alternative treatment to prevent thromboembolism for patients who have contraindications to long-term oral anticoagulation (OAC) therapy and has been proven safe and effective for preventing atrial fibrillation (AF)-related stroke, systemic embolism, and bleeding events (1, 2) The “one-stop” procedure, combined catheter ablation and LAAC in a single procedure based on shared operative approaches, could restore sinus rhythm to relieve AF symptoms and prevent stroke in high-risk patients to avoid the need of long-term OAC and reduce the risk of bleeding events (3).

Transesophageal echocardiography (TEE) was performed routinely to guide the LAAC procedure with the need for general anesthesia and endotracheal intubation. Intracardiac echocardiography (ICE)-guided procedure is performed under local anesthesia, which could reduce the risk of esophageal injury and complications of general anesthesia. Previous studies described that ICE had emerged as an alternative to TEE for LAAC device guidance (4, 5). However, few studies compared the use of ICE vs. TEE in terms of the “one-stop” procedure. We hypothesized that ICE could provide comparable measurements of the left atrial appendage (LAA) with TEE and could reduce the complications compared with TEE. In this study, we aimed to assess the efficacy and safety of the “one-stop” procedure guided by ICE or TEE in our single center.

## Methods

### Study population

We prospectively enrolled 302 consecutive patients with documented non-valvular AF who underwent the “one-stop” procedure in our single center between January 2021 and October 2022. The indications for the procedure are as follows: age more than 18 years; patients with paroxysmal or persistent non-valvular AF; refractory to antiarrhythmic drugs; CHA2DS2-VASc score ≥2 in men or ≥3 in women, plus one of the following situations, e.g., high risk of bleeding (HAS-BLED score ≥ 3), contraindications for long-term OAC therapy (patients with bleeding events or thromboembolic events under OAC), intolerance or refusal to take OAC, and preference for Watchman device implantation as an alternative to long-term OAC (6, 7). The exclusion criteria are as follows: thrombus formation in LAA; left ventricular ejection fraction (LVEF) <30% (8); patients with hyperthyroidism; and patients with rheumatic mitral valve disease and/or an artificial heart valve. This study was approved by the ethics board of the General Hospital of Northern Theater Command, Shenyang, China. The patients signed an informed written consent form.

### Preoperative assessment

In patients on warfarin with low time in the INR therapeutic range (TTR < 70%), switching to a non-vitamin K antagonist AOC (NOAC) is recommended according to the 2020 European Society of Cardiology (ESC) guidelines. Continuous NOACs were used in all patients in our study for at least 3 weeks before the procedure. All patients underwent laboratory tests, 12-lead electrocardiography, and cardiac computed tomography angiography. TEE was performed within 2 days before the procedure to exclude LAA thrombus and assess the morphology and size of LAA from different views (0°, 45°, 90°, and 135°). Transthoracic echocardiography (TTE) was performed routinely to evaluate heart structure, cardiac function, and the presence of pericardial effusion.

### Procedure workflow

#### Operation team

Radiofrequency catheter ablation (RFCA), LAAC, and ICE were performed by four experienced operators with more than 100 procedures, whereas general anesthesia and TEE were conducted by a single experienced anesthetist and sonographer, respectively. The procedure strategy was at the discretion of the physician.

### RFCA procedure

The RFCA was performed under local anesthesia. Two transseptal punctures were performed with the guidance of ICE and/or x-ray images. After that, heparin was administered, and the activated clotting time (ACT) was maintained at 250–350 s as normal and 300–350 s in the case of left atrial spontaneous echocardiographic contrast (LASEC). Under the guidance of a CARTO 3 mapping system (Biosense Webster, Diamond Bar, CA, USA), circumferential pulmonary vein isolation (CPVI) was performed under the guidance of ablation index in all patients. Additional line ablation and non-pulmonary vein (PV) foci ablation were performed if necessary. The endpoint of RFCA was the elimination of the local PV potential and bidirectional conduction block of the ablation lines. Sinus rhythm was achieved by either ablation or cardioversion.

### LAAC procedure

LAAC was performed subsequently if the absence of thrombus in the left atrium (LA) had been proved by ICE or TEE. The diagnostic criteria for thrombus in the LAA are as follows: well-circumscribed, more or less echo refractile mass with a different texture from the atrial wall and uniform consistency, and often pedunculated, as observed in LAA from multi-angle scanning (9, 10). The diagnostic criteria of ICE or TEE for thrombus in the LAA are the same, and the diagnosis is confirmed by experienced operators and sonographers. Otherwise, the procedure would be interrupted, and OAC was administrated until the thrombus disappeared. The WATCHMAN occluder (Boston Scientific, Natick, MA, USA) was implanted under ICE guidance by local anesthesia (ICE group) or under TEE guidance by general anesthesia (TEE group). The ICE catheter was advanced into the LA by the same pathway as the ablation procedure, whereas the TEE probe was advanced into the mid-esophageal level. LAA ostial width and depth were measured in three views on ICE (left superior PV view, LA home view, and mitral annular view), which were close to 45°, 90°, and 135° on TEE. The ostium was measured from the left circumflex or mitral valve annulus to a point 2 cm below the LA ridge. A pigtail catheter was advanced into the LAA, and angiograms were obtained at the right anterior oblique at 30° and caudal at 20° to assess LAA ostial diameter, depth, and morphology. The device was released after device stability and position were verified by position, anchor, size, and seal (PASS) criteria under the guidance of TEE or ICE. Once released, immediate TEE or ICE was performed to reconfirm the position, residual flow, and compression ratio of the device from 45°, 90°, and 135°. Intraprocedural thrombus formation in LAA was defined as the absence of thrombus in LA proved by preoperative TEE within 2 days before the procedure, whereas thrombus in LA was detected by ICE or TEE after the ablation (ICE group) or general anesthesia (TEE group) and before the LAAC procedure. The turnaround time was defined as the time from the start of the transseptal puncture to out of room. Complications were continuously monitored by ultrasonography or fluoroscopy throughout the procedure. The procedure success was defined as no peri-device leak (PDL) >5 mm on ultrasound images, no device-related complications, and no procedure-related complications (11). In the patients who underwent the combined procedure successfully, the measurements of the LAA ostium by different modalities from 135° were matched with the actual device size. Matched measurements were defined as device compression ranging between 10% and 30%, undersized measurements of the LAA ostium were defined as device compression >30%, and oversized measurements of LAA ostium were defined as device compression <10%.

### Follow-up

AF recurrence was defined according to the symptoms of the patients and/or documented AF/atrial flutter/atrial tachycardia lasting more than 30 s after the 3-month blanking period. TTE was performed to confirm the position of the implantation and exclude device embolization and pericardial effusion the next day after the procedure. TEE was performed 3 months after the procedure to assess the device position, PDL, and device-related thrombosis (DRT). Outpatient and transtelephonic follow-ups were carried out to evaluate thromboembolic and bleeding events. Antiarrhythmic drugs and anticoagulation therapies were recommended at the original dose for at least 3 months. Dual antiplatelet therapy for another 3 months and then life-long aspirin were recommended if no DRT or PDL >5 mm was detected by TEE (12). Otherwise, the anticoagulation therapy was continued, and TEE was performed after the next 3 months.

### Statistical analysis

The continuous variables were reported as means ± standard deviations. Categorical variables were expressed as proportions and compared using the chi-square test. The *t-*test was used to compare continuous data with normal distribution, and the rank sum test was used to compare the two-sample mean of the non-normal distribution. The agreement between different modalities was assessed with intraclass correlation coefficients (ICC). *P-*value < 0.05 was considered statistically significant. Statistical analyses were performed using SPSS 26.0 (IBM, Armonk, NY, USA).

## Results

### Patients’ characteristics

A total of 302 consecutive patients with non-valvular AF underwent the “one-stop” procedure between January 2021 and October 2022 in our single center, including 193 guided by ICE under local anesthesia and 109 guided by TEE under general anesthesia. There were no significant differences between the two groups in terms of age, gender, body mass index, type of AF, HAS-BLED score, comorbidities, left atrial size, left ventricular size, LVEF, LASEC, and LAA flow velocity (*P* > 0.05). The CHA2DS2-VASc score was higher in the ICE group than in the TEE group (3.87 ± 1.60 vs. 3.41 ± 1.82, *P* = 0.027). The baseline characteristics are presented in Table 1.

**Table 1:** Baseline characteristics of the study population.

	ICE (*n* = 193)	TEE (*n* = 109)	*P*-value
Age (years)	65.02 ± 8.47	64.23 ± 7.75	0.207
Male	109 (56.48)	69 (63.30)	0.247
BMI (kg/m^2^)	25.93 ± 3.16	25.43 ± 2.86	0.256
Paroxysmal atrial fibrillation	95 (49.22)	44 (40.37)	0.138
CHA2DS2-VASc score	3.87 ± 1.60	3.41 ± 1.82	0.027
HAS-BLED score	2.19 ± 1.15	2.07 ± 1.28	0.372
Hypertension	114 (59.07)	71 (65.14)	0.298
Diabetes	61 (31.61)	27 (24.77)	0.209
Coronary artery disease	58 (30.05)	26 (23.85)	0.248
Previous TIA or stroke	98 (50.78)	46 (42.20)	0.152
Previous bleeding events	4 (2.07)	4 (3.67)	0.648
Chronic heart failure	79 (40.93)	39 (35.78)	0.378
HCM	4 (2.07)	3 (2.75)	0.706
NT-proBNP (ng/L)	993.80 ± 941.75	867.97 ± 947.62	0.369
Left atrial size (mm)	41.68 ± 5.32	42.07 ± 5.74	0.606
Left ventricular size (mm)	50.02 ± 5.16	49.80 ± 4.73	0.760
Left ventricular ejection fraction (%)	0.58 ± 0.09	0.58 ± 0.08	0.773
LASEC (%)	84 (43.52)	41 (37.61)	0.317
Left atrial appendage flow velocity (m/s)	0.36 ± 0.16	0.37 ± 0.17	0.724

Values are mean ± standard deviation or *n* (%).

BMI, body mass index; HCM, hypertrophic cardiomyopathy; NT-proBNP, N-terminal pro-B-type natriuretic peptide; TIA, transient ischemic attack.

### Procedural characteristics

**Figure 1:**
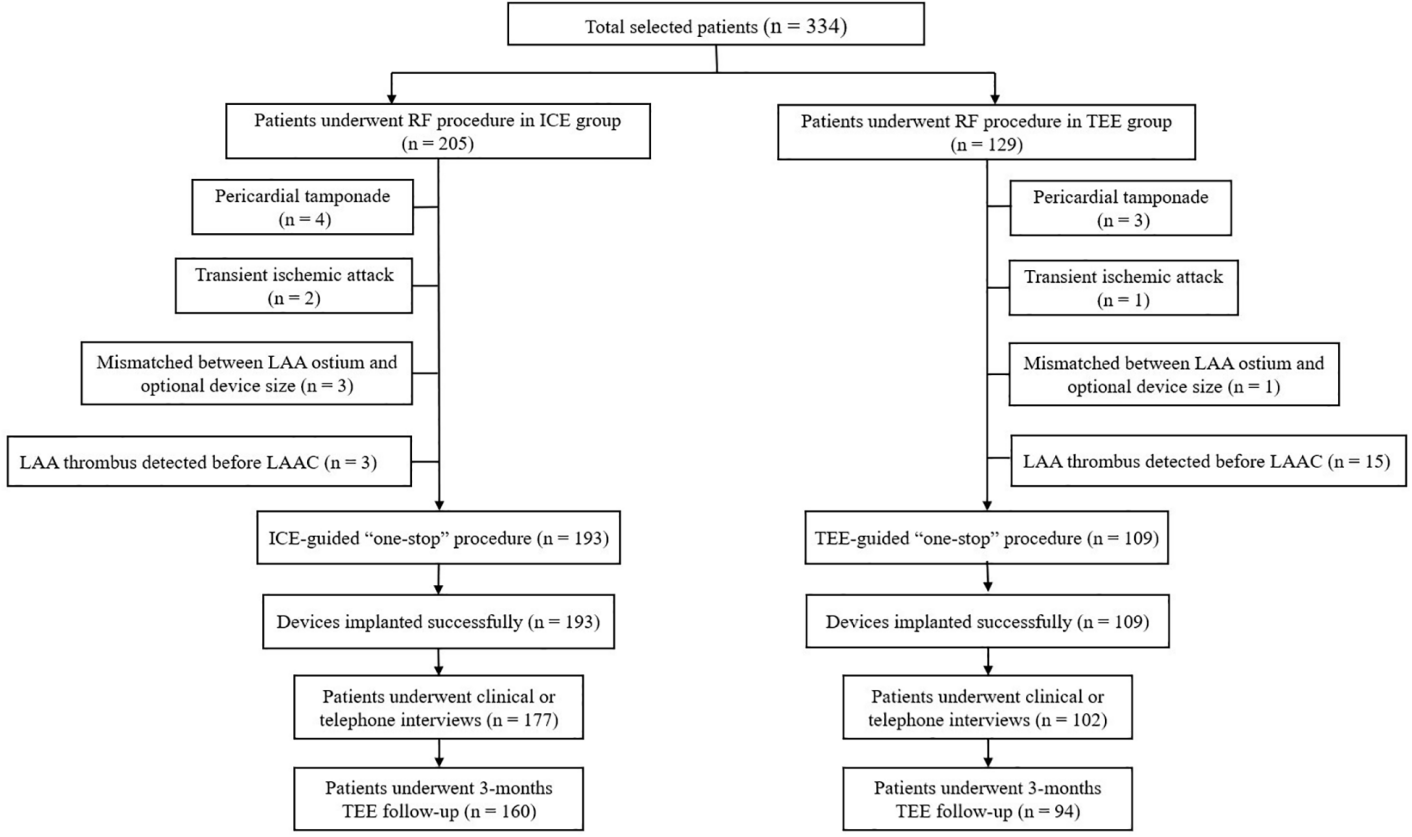
Study flowchart of the “one-stop” procedure in the ICE and TEE groups.

**Figure 2:**
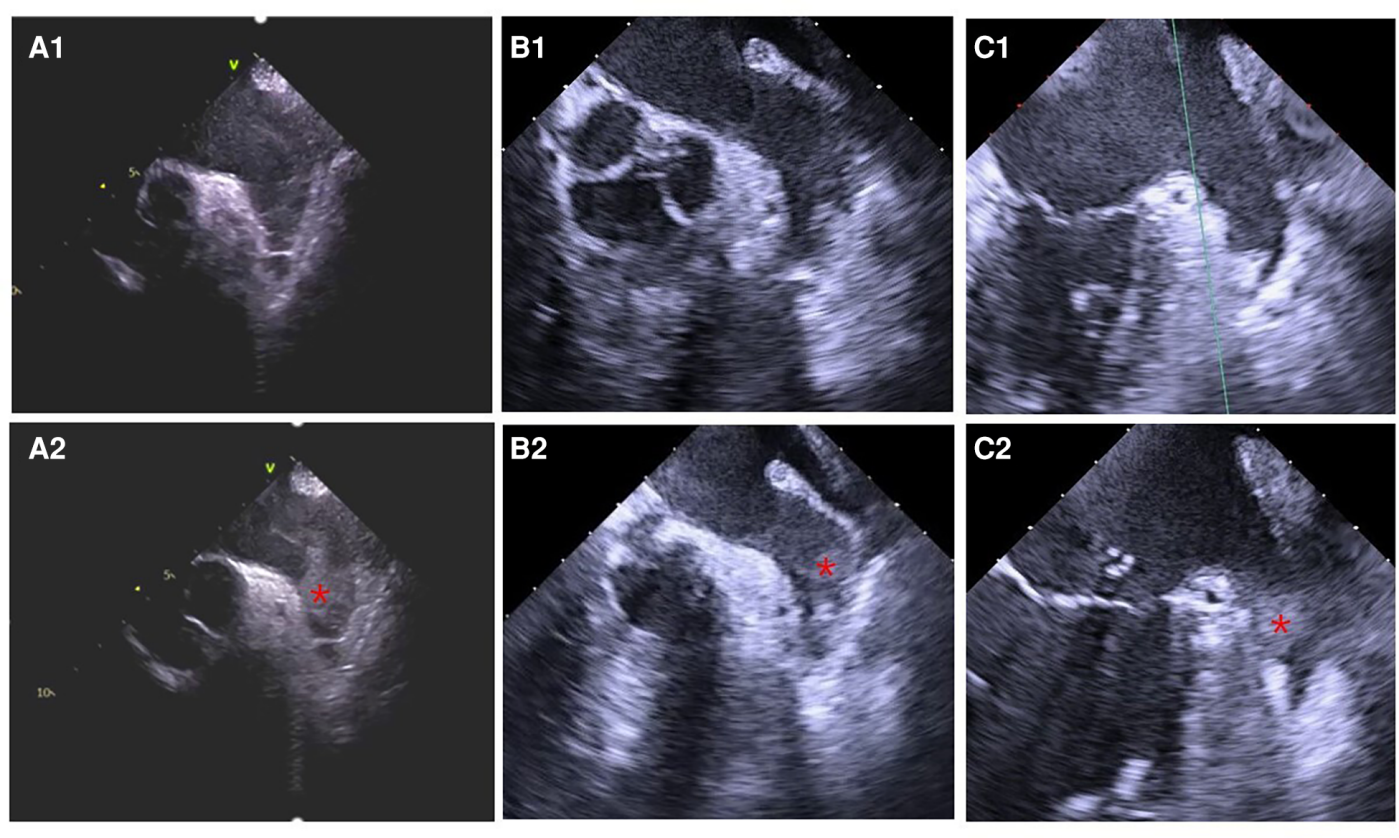
Thrombus formation in LAA detected by ultrasound before the LAAC procedure. (**A1**) ICE, (**B1**) TEE, and (**C1**) TEE: LASEC detected before thrombus formation in LAA. (**A2**) ICE, (**B2**) TEE, and (**C2**) TEE: thrombus formation in LAA identified before LAAC. Informed consent had been signed by the patients to publish the images.

The study flowchart is represented in Figure 1. Nine patients (4.39%) in the ICE group and 19 patients (14.73%) in the TEE group did not undergo LAAC due to complications during RF or thrombus formation in LAA. Intraprocedural thrombus formation in LAA was observed in 3 (1.46%) patients in the ICE group and 15 (11.63%) patients in the TEE group (*P* < 0.05) when ultrasonic examinations were performed before the LAAC procedures (Figure 2). Detailed baseline characteristics of the 15 patients with LAA thrombus in the TEE group are presented in Table 2. Anticoagulation status was confirmed, and additional heparin was administered to achieve an ACT at 300–350 s. Reassessment with ultrasound documented no change in the thrombus state in the two groups. Three patients in the ICE group and one patient in the TEE group failed to receive LAAC due to a mismatch between the oversized LAA ostium and optional device size. All PVs were successfully isolated. Additional line ablation was performed in 27 patients (13.99%) in the ICE group and 19 patients (17.43%) in the TEE group (*P* = 0.424). Cardioversion after CPVI was performed in 63 patients (32.64%) in the ICE group and 39 patients (35.78%) in the TEE group, respectively (*P* = 0.580). Sinus rhythm was present in all patients after ablation. There was no significant difference in RFCA procedure time between the two groups [(64.36 ± 23.13) min in the ICE group vs. (59.97 ± 21.13) min in the TEE group, *P* = 0.097]. No significant difference in the thickness of the ridge region after RFCA was seen in the two groups. LAA morphologies and lobes were comparable between the two groups. The Watchman devices were implanted successfully in all the cases in both groups. There was no difference in device diameters between the two groups. Device compression from different views was comparable between the ICE and TEE groups. The proportions of cases presented with residual flow ≤3, >3, and ≤5 mm and had no difference between the two groups. During the procedure, no residual flow >5 mm was observed in either group. The devices were retrieved due to unsatisfactory device compression in 18 patients (9.33%) in the ICE group and nine patients (8.26%) in the TEE group (*P* > 0.05). Total fluoroscopy time in the ICE group was shorter than that in the TEE group [(21.56 ± 8.86) vs. (25.43 ± 8.83) min, *P* = 0.002]. The fluoroscopy dose used in the ICE group was less compared with that in the TEE group [(430.64 ± 278.74) vs. (466.63 ± 277.49) mGy, *P* = 0.015]. The total “one-stop” turnaround time and LAAC procedure time in the ICE group were significantly shorter than those in the TEE group [(126.30 ± 32.43) min vs. (144.18 ± 26.17) min, (60.20 ± 20.65) min vs. (73.94 ± 16.04) min, *P *< 0.05). Procedural characteristics in the TEE and ICE groups are presented in Table 3.

**Table 2:** Baseline characteristics of the 15 patients with LAA thrombus in the TEE group.

	Patients without LAA thrombus (*n* = 110)	Patients with LAA thrombus (*n* = 15)	*P*-value
Age (years)	64.17 ± 7.74	67.13 ± 8.54	0.174
Male	70 (63.64)	9 (60)	0.784
Paroxysmal atrial fibrillation	44 (40)	4 (26.67)	0.319
CHA2DS2-VASc score	3.42 ± 1.82	4.07 ± 1.94	0.205
HAS-BLED score	2.08 ± 1.27	2.00 ± 0.85	0.758
Hypertension	72 (65.45)	12 (80)	0.405
Diabetes	27 (24.55)	7 (46.67)	0.134
Coronary artery disease	27 (24.55)	6 (40)	0.336
Previous TIA or stroke	47 (42.73)	7 (46.67)	0.773
Previous bleeding events	4 (3.64)	1 (6.67)	0.478
Chronic heart failure	39 (35.45)	8 (53.33)	0.180
Left atrial size (mm)	42.11 ± 5.73	45.17 ± 3.83	0.075
Left ventricular ejection fraction (%)	0.58 ± 0.08	0.56 ± 0.09	0.594
LASEC (%)	42 (38.18)	10 (66.67)	0.036
Left atrial appendage flow velocity (m/s)	0.37 ± 0.17	0.23 ± 0.06	<0.001

**Table 3:** Procedural characteristics and outcomes.

	ICE (*n* = 193)	TEE (*n* = 109)	*P-*value
Number of atrial septal puncture sites			
1	45 (23.32)	22 (20.18)	0.529
2	148 (76.68)	87 (79.82)	0.529
CPVI plus linear ablation	27 (13.99)	19 (17.43)	0.424
Cardioversion	63 (32.64)	39 (35.78)	0.580
RFCA procedure time (min)	64.36 ± 23.13	59.97 ± 21.13	0.097
Thickness of the ridge region	5.38 ± 2.30	6.04 ± 2.13	0.06
Left atrial appendage morphology (*n*, %)			
Chicken wing	46 (23.83)	24 (22.02)	0.719
Non-chicken wing	147 (76.17)	85 (77.98)	0.719
Left atrial appendage lobes (*n*, %)			
1	85 (44.04)	45 (41.28)	0.642
2	99 (51.30)	59 (54.13)	0.636
≥3	9 (4.66)	5 (4.59)	0.976
Device diameter (*n*, %)			
21 mm	11 (5.70)	11 (10.09)	0.158
24 mm	28 (14.51)	19 (17.43)	0.501
27 mm	82 (42.49)	37 (33.94)	0.145
30 mm	54 (27.98)	28 (25.69)	0.667
33 mm	18 (9.32)	14 (12.85)	0.340
Device compression (%)			
45°	22.01 ± 5.20	21.49 ± 3.99	0.447
90°	22.07 ± 5.18	22.37 ± 3.83	0.661
135°	22.65 ± 5.24	21.70 ± 3.98	0.175
Residual flow			
0 mm	183 (94.82)	99 (90.82)	0.180
≤3 mm	10 (5.18)	9 (8.26)	0.290
>3 mm, ≤5 mm	0 (0)	1 (0.92)	0.361
Number of devices used			
1	175 (90.67)	100 (91.74)	0.754
≥2	18 (9.33)	9 (8.26)	0.754
Fluoroscopy time (min)	21.56 ± 8.86	25.43 ± 8.83	0.001
Fluoroscopy dose (mGy)	430.64 ± 278.74	466.63 ± 277.49	0.015
LAAC procedure time (min)	60.20 ± 20.65	73.94 ± 16.04	0.001
Turnaround time	126.30 ± 32.43	144.18 ± 26.17	0.006

### Measurements of LAA dimension by preoperative and intraoperative modalities

**Figure 3:**
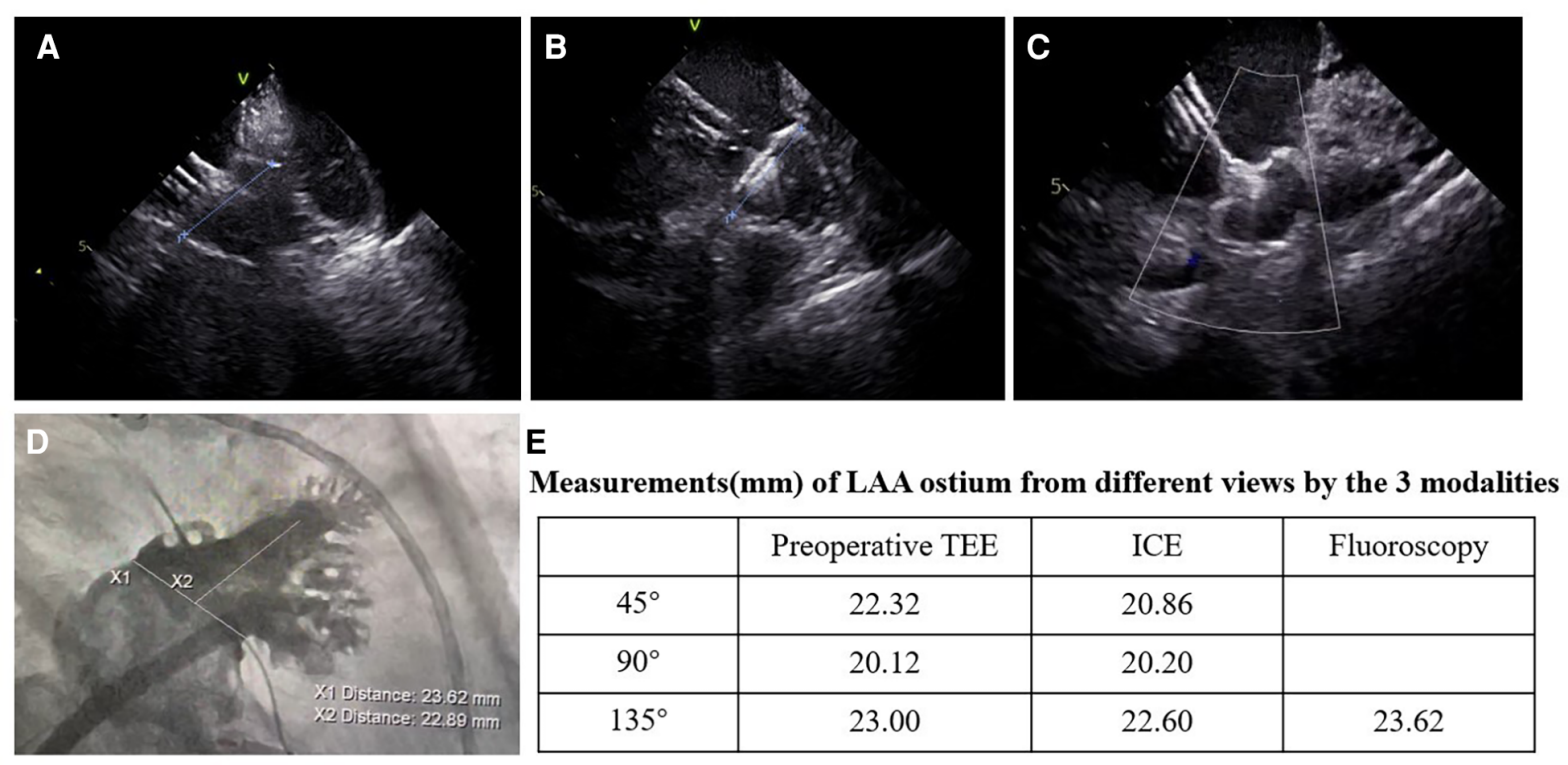
Images of the LAAC with the WATCHMAN device from different views in the ICE-guided procedure. (**A–C**) Assessment of device position by ICE; (**A**) ICE from left superior pulmonary vein view; (**B**) ICE from LA home view; (**C**) ICE from mitral annular view; (**D**) images of fluoroscopy at right anterior oblique at 30° and caudal at 20°; (**E**) measurements of the LAA ostium from the three views by preoperative TEE, ICE, and fluoroscopy.

**Figure 4:**
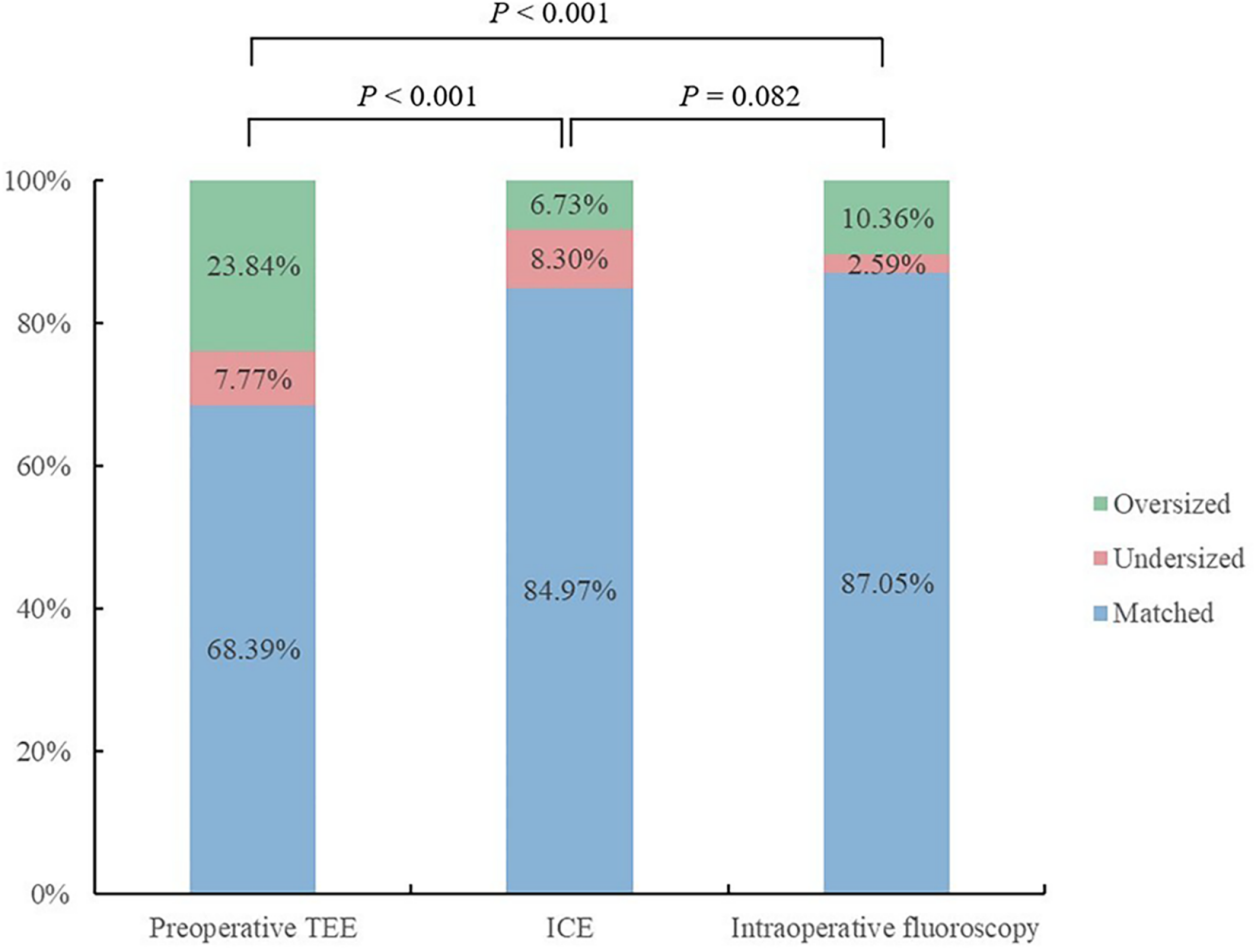
Outcomes of the measurements of the LAA ostium by different modalities from 135° matched with the actual device size.

ICC was used to assess agreement between different modalities in the ICE group (Table 4). The results showed that all modalities were well correlated (*P* < 0.001). The agreement between ICE from 135° and fluoroscopy was higher with an ICC of 0.797 than that between TEE from 135° and fluoroscopy and those between preoperative TEE and ICE from different angles. In the 193 patients who underwent the “one-stop” procedure successfully in the ICE group, LAA dimensions measured by fluoroscopy best matched the actual size of the implanted device. Matched measurements were more often obtained by fluoroscopy than preoperative TEE (*P* < 0.05). There was no significant difference in matched cases between ICE and fluoroscopy measurements (*P* = 0.082) (Figures 3, 4).

**Table 4:** ICC of LAA ostium measured by different modalities in the ICE group.

	ICC	95% CI		*F*-value	*P*-value
		Lower limit	Upper limit		
45°					
ICE—preoperative TEE	0.652	0.459	0.776	2.872	<0.001
90°					
ICE—preoperative TEE	0.639	0.439	0.768	2.771	<0.001
135°					
ICE—preoperative TEE	0.656	0.466	0.779	2.910	<0.001
ICE (135°)—fluoroscopy	0.797	0.700	0.863	4.929	<0.001
Preoperative TEE (135°)—fluoroscopy	0.569	0.329	0.724	2.323	<0.001

### Perioperative complications

Pericardial tamponade occurred in four (2.07%) patients in the ICE group and three (2.75%) patients in the TEE group (*P* = 0.706). Of these seven patients, five were successfully treated by pericardiocentesis and two were resolved by cardiac surgery. Two (1.04%) patients in the ICE group and one (0.92%) patient in the TEE group experienced transient ischemic attack with transient limb movement disorder (*P* > 0.05). The computed tomography brain scanning was negative for ischemic lesions, and all patients were discharged without any clinical sequelae. No procedure-related stroke occurred in the two groups. The occurrence of vagal reflex and pseudoaneurysm showed no statistical difference between the two groups. The pseudoaneurysm was successfully cured by compression under the guidance of ultrasound. Postoperative esophagus discomfort, nausea and vomiting, and hypotension were more often seen in the TEE group (*P* < 0.001). No air embolism, device embolization, or death was recorded in the two groups. Detailed information on perioperative complications is given in Table 5.

**Table 5:** Perioperative complications.

	ICE (*n* = 193)	TEE (*n* = 109)	*P-*value
Pericardial tamponade	4 (2.07)	3 (2.75)	0.706
TIA	2 (1.04)	1 (0.92)	1.000
Vagal reflex	44 (22.80)	19 (17.43)	0.270
Femoral pseudoaneurysm	2 (1.04)	0 (0)	0.537
Postoperative esophagus discomfort	0 (0)	11 (10.09)	<0.001
Postoperative nausea and vomiting	2 (1.04)	21 (19.27)	<0.001
Postoperative hypotension	2 (1.04)	14 (12.84)	<0.001

### Follow-up

#### Arrhythmia recurrence and clinical events

A total of 177 (91.71%) patients in the ICE group and 102 (93.58%) patients in the TEE group were followed up by clinical and telephone interviews for an average time of 15.67 ± 3.76 months and 16.49 ± 2.88 months, respectively (*P* = 0.075). In addition, 138 (77.97%) patients in the ICE group and 77 (75.49%) patients in the TEE group exhibited sinus rhythm (*P* = 0.636). In total, 14 (7.91%) patients in the ICE group and seven (6.86%) patients in the TEE group underwent repeated catheter ablation. Ischemic stroke was observed in two patients (1.96%) at 12 days and 6 months post-procedure in the TEE group. Unexplained delayed cardiac tamponade occurred in one patient (0.56%) in the ICE group and one patient (0.98%) in the TEE group. Both were treated successfully by pericardiocentesis. No major bleeding or systemic embolism was observed in either group.

### TEE follow-up

TEE follow-up was performed in 160/193 (82.90%) patients in the ICE group and 94/89 (86.24%) patients in the TEE group (*P* = 0.446) at 3 months postoperatively. LAA occlusion was complete in 143 (89.38%) patients in the ICE group and 79 (84.04%) patients in the TEE group (*P* = 0.216). A total of 11 (6.87%) patients in the ICE group and 10 (10.64%) patients in the TEE group presented with PDL ≤3 mm, whereas six (3.75%) patients in the ICE group and five (5.32%) patients in the TEE group presented with PDL >3 mm and ≤5 mm, respectively. DRT was detected in one patient in the ICE group and one patient in the TEE group due to early discontinuation of OAC (0.63% vs. 1.06%; *P* > 0.05) and was resolved by prolonged anticoagulation therapy for another 3 months. Iatrogenic atrial septal defects (iASDs) were detected in 44 (27.50%) patients in the ICE group and 23 (24.47%) patients in the TEE group (*P* = 0.597). Two residual iASDs were detected in six (3.75%) patients in the ICE group and three (3.19%) patients in the TEE group (*P* > 0.05). There was no significant difference in the mean size of iASDs between the two groups [(3.97 ± 1.62) mm vs. (4.14 ± 1.57) mm; *P* = 0.735)].

### Post-procedural antithrombotic therapy management

OCA was discontinued in 153 patients (95.63%) in the ICE group and 88 patients (93.62%) in the TEE group at 3 months postoperatively and substituted with dual antiplatelet therapy. Thirteen patients remained on OCA for 6 months due to DRT or PDL >3 mm [seven (4.37%) patients in the ICE group and six (6.38%) patients in the TEE group]. Four patients in the ICE group and four patients in the TEE group remained on dual antiplatelet therapy due to coronary stents, whereas 246 of the 254 patients were prescribed aspirin only 6 months postoperatively. Follow-up results are represented in Table 6.

**Table 6:** Follow-up data.

	ICE *n* = 193)	TEE (*n* = 109)	*P-*value
Clinical and telephone interviews	177 (91.71)	102 (93.58)	0.557
Average time of follow-up (months)	15.67 ± 3.76	16.49 ± 2.88	0.075
Sinus rhythm	138 (77.97)	77 (75.49)	0.636
Arrhythmia recurrence			
Paroxysmal AF	23 (12.99)	14 (13.73)	0.862
Persistent AF	10 (5.65)	7 (6.86)	0.683
Atrial flutter	6 (3.39)	4 (3.92)	1.000
Redo ablation	14 (7.91)	7 (6.86)	0.750
Stroke	0 (0)	2 (1.96)	0.133
Delayed cardiac tamponade	1 (0.56)	1 (0.98)	1.000
TEE follow-up at 3 months	160 (82.90)	94 (86.24)	0.446
Peri-device leak			
None	143 (89.38)	79 (84.04)	0.216
≤3 mm	11 (6.87)	10 (10.64)	0.293
>3 mm, ≤5 mm	6 (3.75)	5 (5.32)	0.784
Device-related thrombosis	1 (0.63)	1 (1.06)	1.000
Residual iASDs	44 (27.50)	23 (24.47)	0.597
Number of residual iASDs			
1	38 (23.75)	20 (21.28)	0.650
2	6 (3.75)	3 (3.19)	1.000
Size of the iASD	3.97 ± 1.62	4.14 ± 1.57	0.735

## Discussion

The one-stop procedures may be performed in different sequences with catheter ablation (CA) first or LAAC first. The LAAC success rate after CA in the one-stop procedure was very high, which was similar to the success rate of LAAC alone, as reported in the EWOLUTION study (13). The LAAC procedures were all performed after RFCA in our center, and the success rate of LAAC was 100%, whether guided by ICE or TEE. It had been reported that the edematous ridges had a considerable impact on the LAAC procedure performed by different types of occlusion devices (14). For the CA-first procedure, the acute tissue edema induced by RF heating could lead to inappropriate decisions on the size of the device and increase the risk of PDL after the edema subsided. In our study, the thickness of the ridge region was 5.38 ± 2.30 mm in the ICE group and 6.04 ± 2.13 mm in the TEE group. A total of 11 (6.87%) patients in the ICE group and 10 (10.64%) patients in the TEE group presented with residual flow ≤3 mm, whereas 6 (3.75%) patients in the ICE group and 5 (5.32%) patients in the TEE group presented with PDL >3 mm and ≤5 mm, respectively. The incidence of residual PDL was less than that in the observational studies (15, 16), which may be due to the little impact of edematous ridges on the Watchman device implanted in the LAA inner ostium.

TEE performed by a dedicated sonographer is the gold standard to guide LAAC procedure, but it may pose a risk of esophageal injury (17). General anesthesia and endotracheal intubation are necessary to avoid patient motion and discomfort in TEE-guided procedures. ICE-guided “one-stop” procedure was performed under local anesthesia, saving anesthetic time and avoiding delayed recovery of consciousness. In this study, the total “one-stop” turnaround time and LAAC procedure time in the ICE group were significantly shorter than those in the TEE group. ICE instead of TEE also reduced the risk of esophageal injury. As detected in this study, the rates of postoperative esophagus discomfort, nausea, and vomiting were more often seen in the TEE group than those in the ICE group (*P* < 0.05).

According to “Left atrial appendage interventions for thromboembolism prevention in patients with atrial fibrillation: current knowledge and recommendation (2019)” in China (8), LAAC is not recommended in patients with LVEF < 30% or New York Heart Association class IV that have not been corrected yet. Low LVEF has been reported to be associated with PDL and DRT (18, 19).

Despite the lower CHA2DS2-VASc score, intraprocedural thrombus formation in LAA was more often observed in the TEE group than in the ICE group when ultrasonic examinations were performed before the LAAC procedures. In this study, the proportion of patients presented with LASEC was 43.52% in the ICE group and 37.61% in the TEE group. Impaired systolic function of the LAA was more often presented in these patients. Moreover, the need for preoperative fasting and general anesthesia resulted in intraprocedural hypotension, which increased the risk of intraprocedural thrombus formation in LAA in the TEE group. Some cardiologists have advocated for fluoroscopy-guided LAAC without echocardiography guidance, which appears feasible and safe (20, 21). In another study (22), to shorten the duration of TEE probe placement, TEE was performed at the end of the procedure to confirm the position and residual flow of the occluder device. As mentioned in our results, we believed that echocardiography was necessary to rule out the intraoperative thrombus and reduce the risk of perioperative stroke during the “one-stop” procedures.

Fluoroscopy is typically used to obtain transseptal access to the LA and guide the LAAC procedure. However, radiation exposure, which was considered a silent complication, is harmful to the health of operators and patients. Previous studies have described that radiation exposure could increase the risk of dermatologic injury, musculoskeletal injury, fatal malignancy, and genetic defects (23). The ALARA principle has been promoted to minimize fluoroscopy exposure in electrophysiologic procedures (24). ICE allows high-resolution real-time visualization to provide an ideal transseptal puncture site, monitor the ablation catheter position, and warrant real-time continuous monitoring for potential intraprocedural complications (25, 26). Considering the advantage of ICE, the total fluoroscopy time and fluoroscopy dose in the ICE group were less than those in the TEE group (*P* = 0.002, *P* = 0.015) in this study, which was consistent with previous studies (27). Zero-fluoroscopy “one-stop” procedures have become feasible using a combination of ICE and three-dimensional electroanatomical mapping systems (28, 29). Pericardial effusion could be detected by ICE before hemodynamic changes and be intervened with anticoagulation reversal and pericardiocentesis to prevent the occurrence of cardiac tamponade (25). The incidence of pericardial tamponade had no significant difference between the two groups, while the detection by ICE performed by interventional cardiologists was more convenient and timely. ICE has been proven safe and feasible as an alternative to TEE in guiding LAAC procedures in multiple studies (25, 30, 31) to assess the dimensions and shapes of LAA and guide device placement. In this study, LAA dimensions measured by fluoroscopy were best matched with the actual size of the implanted device in both groups. There was no significant difference in matched cases between ICE and fluoroscopy measurements in the ICE group. The ICE catheter delivered into the LA allows close and multi-angle scanning of the LAA, avoiding the limitations of TEE application in patients with cardiac transposition and atrial appendage variations (32, 33).

It has been hypothesized that the increased rate of iASD associated with ICE guidance may be due to the shear stress caused by crossing of the ICE probe into the LA and subsequent manipulation, in addition to the standard crossing of the delivery system (34). In our study, two transseptal punctures were commonly performed in the “one-stop” procedure to avoid interaction with the LAAC sheath and obtain smaller iASD for each. TEE follow-up at 3 months after the procedure showed no significant difference in the incidence or size of iASD between the ICE and TEE groups. No patients suffered from iASD-related consequences.

In a meta-analysis, the main findings were that there was no significant difference in acute procedural success, complications, fluoroscopy time, and total procedure time between TEE vs. ICE-guided LAAC. The study emphasized that it required a certain level of expertise to guarantee a high success rate and few complications (35). In our study, RFCA, LAAC, and ICE were all performed by experienced operators. Other studies have shown similar results to ours. Hemam et al. (5) reported that an ICE-guided WATCHMAN implant was safe, feasible, and comparable in cost to TEE during LAAC with a Watchman device but avoided GA and expedited procedure turnaround. Gianni et al. described that ICE-guided LAAO with Watchman FLX was safe and feasible, with a significant reduction in procedural time and potential reduction in fluoroscopy dose compared to TEE (36).

## Limitations

Our study had several limitations. First, this was a single-center observational study that enrolled a limited number of patients. Further long-term, multicenter, and large-sample studies are required to confirm the conclusion. Second, the measurements of LAA dimensions by various modalities were analyzed by different operators, which may confuse the results. Third, the procedures were performed by operators with extensive experience in using ICE and TEE, which may affect the reproducibility of the results. Fourth, patients with low EF < 30% were excluded from the study. Finally, the WATCHMAN device is the only single LAAC device used in our study, and the results of this study cannot be generalized.

## Conclusion

The ICE-guided “one-stop” procedure was safe and feasible with less radiation exposure, shorter turnaround time, and fewer complications and intraoperative thrombus formations than TEE. ICE offered accurate measurements in the LAA dimension during LAAC. Echocardiography was necessary during the “one-stop” procedures to rule out the intraoperative thrombus.

## Data Availability

The raw data supporting the conclusions of this article will be made available by the authors, without undue reservation.
